# Social support and posttraumatic growth in a longitudinal study of people living with HIV: the mediating role of positive affect

**DOI:** 10.1080/20008198.2017.1412225

**Published:** 2017-12-18

**Authors:** Marcin Rzeszutek

**Affiliations:** ^a^ Faculty of Psychology, University of Warsaw, Warsaw, Poland

**Keywords:** HIV/AIDS, posttraumatic growth, social support, positive affect, negative affect

## Abstract

**Background:** Psychological research in people living with HIV (PLWH) has been dominated by studies on the negative consequences of HIV infection. However, recently, positive changes following the diagnosis of HIV have been examined, namely the phenomenon of posttraumatic growth (PTG).

**Objectives**: The aim of this one-year longitudinal study was to investigate the level of PTG and its relationship with social support dimensions (perceived support, need for support, actually received support) and positive and negative affect among PLWH. Specifically, this study explored the mediating role of positive and negative affect in the link between social support and PTG.

**Method:** Participants filled out the following psychometric tools: Posttraumatic Growth Inventory (PTGI), Berlin Social Support Scales (BSSS) and Positive and Negative Affect Schedule (PANAS-X). Three assessments were performed. Altogether, 129 patients were recruited for the first assessment, 106 patients participated in the second assessment and 82 participants (63.6%) out of the initial sample of 129 participated in all three assessments.

**Results**: The positive relationship between all examined social support dimensions and PTG was entirely mediated by positive affect. There was no association between negative affect and PTG. Selected socio-medical covariates (sex, employment, higher education, being in a stable relationship and HIV/AIDS status) were related to social support and PTG among participants.

**Conclusions**: This study points to the need for more research on positive aspects of HIV/AIDS, notably PTG. More specifically, interventions focused on enhancement and sustainment of positive affect among PLWH should be an adjunct to traditional mental health screening among this patient group.

## Apoyo social y crecimiento postraumático en un estudio longitudinal de personas que viven con VIH: el papel mediador del afecto positivo

**Planteamiento:** La investigación psicológica en personas que viven con VIH (PLWH, siglas en inglés de *people living with HIV*) ha estado dominada por estudios sobre las consecuencias negativas de la infección por VIH. Sin embargo, recientemente también se han examinado los cambios positivos después del diagnóstico de VIH, a saber, el fenómeno de crecimiento postraumático (CPT).

**Objetivos:** El objetivo de este estudio longitudinal de un año fue investigar el nivel de CPT y su relación con las dimensiones de apoyo social (apoyo percibido, necesidad de apoyo, apoyo realmente recibido) y afecto positivo y negativo entre las PLWH. Específicamente, este estudio exploró el papel mediador de la emoción positiva y negativa en el vínculo entre el apoyo social y el CPT.

**Método:** Los participantes completaron las siguientes herramientas psicométricas: el Inventario de Crecimiento Postraumático (PTGI, siglas en inglés de *Posttraumatic Growth Inventory*), las Escalas de Apoyo Social de Berlín (BSSS, siglas en inglés de *Berlin Social Support Scales*) y el Programa de Afecto Positivo y Negativo (PANAS-X, siglas en inglés de *Positive and Negative Affect Schedule*). Se realizaron tres evaluaciones. En total, se reclutaron 129 pacientes para la primera evaluación, 106 pacientes participaron en la segunda evaluación y 82 participantes (63,6%) de la muestra inicial de 129 participaron en las tres evaluaciones.

**Resultados:** La relación positiva entre todas las dimensiones de apoyo social examinadas y el CPT estuvo completamente mediada por la emoción positiva. No hubo asociación entre emoción negativa y CPT. Las covariantes socio-médicas seleccionadas (sexo, empleo, educación superior, estar en una relación estable y el estado de VIH/SIDA) se relacionaron con el apoyo social y el CPT entre los participantes.

**Conclusiones:** Este estudio señala la necesidad de más investigación sobre los aspectos positivos del VIH/SIDA, en particular, el CPT. Más concretamente, las intervenciones centradas en la mejora y el mantenimiento de la emoción positiva entre las PLWH deberían ser un complemento de las evaluaciones de salud mental tradicionales en este grupo de pacientes.

## HIV 携带者追踪研究中的社会支持和创伤后成长：积极情感的中介作用

**背景：**关于HIV携带者 (PLWH) 的心理研究一直重点关注负面后果。不过，最近研究同时也考察了诊断出HIV 之后的积极改变，即创伤后成长 (PTG) 的现象。

**目标：**这个长达一年的追踪研究旨在考察PLWH 群体中，PTG 水平及其与社会支持维度(知觉到的支持、对支持的需要、实际支持) 以及积极、消极情感之间的关系。本研究尤其探索了积极和消极情感在社会支持和PTG 之间的中介作用。

**方法：**被试填写了一下的心理测量工具：创伤后成长问卷（PTGI）、柏林社会支持量表 (BSSS) 和积极消极情感量表（PANAS-X）。一共进行三次测量，第一次有129名被试，第二次106名，有82名（63.6%）全部参加了三次施测。

**结果：**社会支持的所有维度和PTG 之间正相关都被积极情感中介了。负性情感和PTG 之间没有关联。选出的社会-医学协变量（性别、就业、高教育水平、稳定关系和HIV/AIDS 状况）都和社会支持以及PTG 相关。

**结论：**本研究指出需要更多的关注HIV/AIDS 的积极情感的研究，特别是PTG。更具体的，着重于增加和保持PLWH积极情感的干预方法应该附加在传统的对这个病人群体的心理健康筛查中。

More than two decades ago, Tedeschi and Callhoun (1996) introduced the term posttraumatic growth (PTG), which has since garnered a great deal of interest among researchers and clinicians describing positive changes among survivors of traumatic life events (e.g. Helgeson, Reynolds, & Tomich, 2006; Prati & Pietrantoni, 2009). There is no consensus regarding whether PTG represents an outcome of dealing with traumatic events (Tedeschi & Calhoun, 2004), is an active coping strategy (Tennen & Affleck, 2002) or serves even as a positive illusion (Maercker & Zoellner, 2004). However, most researchers mentioned above report similar changes – including more satisfying interpersonal relationships, finding new life possibilities, greater appreciation of life, openness to spiritual issues and enhanced perception of personal strength – in people exposed to adverse life events. Despite the existence in the literature of other terms describing positive changes after trauma and adversity, such as stress-related growth (Siegel & Schrimshaw, 2000), adversarial growth (Linley & Joseph, 2004) or benefit finding (Danoff-Burg & Revenson, 2005), Tedeschi and Calhoun (2004) distinguished them from PTG. Specifically, PTG occurs with attempts at adapting to an event that is challenging enough to induce a transformation of life and a change in one’s basic life values. Several researchers have observed that this kind of challenging event, which may often trigger growth, is usually a diagnosis accompanied by a struggle with a life-threatening illness (Hefferon, Grealy, & Mutrie, 2009; Sawyer, Ayers, & Field, 2010).

The diagnosis, treatment and subsequent life with a potentially terminal and still highly stigmatized disease, such as HIV/AIDS, constitutes a major stressor, which, according to several authors (e.g. Adewuya et al., 2009; Machtinger, Wilson, Haberer, & Weiss, 2012; Martin & Kagee, 2011; Rzeszutek, Oniszczenko, Żebrowska, & Firląg-Burkacka, 2015; Theuninck, Lake, & Gibson, 2010), meets the criteria of traumatic stressor. The prevalence of HIV-related posttraumatic stress disorder (PTSD) among people living with HIV (PLWH), which stems from receiving a diagnosis of potentially lethal virus in the body and related perceived threat to life, ranges between 30 and 64% (Olley, Zeier, Seedat, & Stein, 2005; Sherr et al., 2011). Nevertheless, some studies also found positive changes following the diagnosis of HIV infection, which comprise the aforementioned dimensions of PTG (Milam, 2004, 2006; Murphy & Hevey, 2013; Rzeszutek, Oniszczenko, & Firląg-Burkacka, 2017; Siegel, Schrimshaw, & Pretter, 2005). For example, Milam (2004) noted that 59% of PLWH experienced some aspects of PTG following their HIV diagnosis, and that those positive changes were negatively associated with the level of depression as well as an improved viral load in this patient group. Luszczynska, Sarkar, and Knoll (2007) observed that PTG was associated with better adherence to treatment among PLWH. Although an increasing number of researchers also highlight positive consequences regarding HIV infection, the nature of PTG among PLWH remains unclear; in particular, researchers investigating PTG among PLWH concentrate primarily on documenting these positive changes, and little attention has been paid to variables that may facilitate or hinder PTG in this patient group (Sawyer et al., 2010). In this study, the relationship between social support and PTG in a one-year longitudinal study of PLWH was examined. In addition, the study investigated whether positive and negative affect may mediate the aforementioned relationship among participants.

Social support, particularly that received from a partner, family or friends (see, ‘supportive others’; Tedeschi & Calhoun, 2004, p. 8) plays a vital role in facilitating PTG by mobilizing cognitive processing after trauma, i.e. fostering ruminative activity (Tedeschi & Calhoun, 2004). The aforementioned positive effect of social support on PTG was observed especially after catastrophic traumatic events, such as an earthquake (Jia, Ying, Zhou, Wu, & Chongde, 2015), flood (Dursun, Steger, Bentele, & Schulenberg, 2016) or terrorist attack (Páez, Basabe, Ubillos, & González, 2007). In addition, perceived social support was related to levels of PTG among people who have recovered from addiction (Haroosh & Freedman, 2017). However, the research on social support and PTG in the context of life-threatening illness remains inconclusive. On the one hand, support seeking (Kent et al., 2013) and received social support (Schroevers, Helgeson, Sanderman, & Ranchor, 2010) were positively related to PTG among some cancer patients. Conversely, the link between social support and PTG was not found among cardiac patients (Sheikh, 2004) and other studies conducted on cancer patients (Sears, Stanton, & Danoff-Burg, 2003). Regarding PLWH, Siegel et al. (2005) observed a positive association between emotional support and PTG among HIV-infected women, while Rzeszutek et al. (2017) observed a direct positive effect of received support on PTG in a longitudinal study. In contrast, Cieslak et al. (2009), in a study of PLWH who survived Hurricane Katrina, found that received support did not predict the total PTG level and that it was positively related only to one PTG dimension, namely, relating to others. Thus, the picture of the link between social support and PTG in cases of life-threatening illness is ambiguous and needs further investigation. In particular, according to Schroevers et al. (2010), these inconsistent results may be attributed to the use of different dimensions of social support and the dominance of cross-sectional studies in this area. In this study, this gap was addressed by analysing various dimensions of social support in relation to PTG in a longitudinal study design.

In Tedeschi and Calhoun’s (2004) PTG model, an appropriate emotion regulation, especially maintaining a high level of positive affect, may indirectly foster growth by managing psychological distress and stimulating the search for meaning after trauma. Linley and Joseph (2004), in a meta-analytic review, noted that PTG is significantly associated with a high intensity of positive affect. More specifically, positive affect turned out to be a mediator variable between PTG and personal resources, namely, self-efficacy (Yu et al., 2014) as well as ruminations and meaning (Boyraz & Efstathiou, 2011). Conversely, maintaining a heightened level of negative affect not only hinders PTG (Boyraz, Horne, & Sayger, 2010), it also deteriorates well-being (Gross & John, 2003). Nevertheless, some studies also indicate that PTG is linked only to a greater level of positive affect and is unrelated to negative affect (Schroevers et al., 2010; Yu et al., 2014). Finally, although the literature on HIV/AIDS is replete with examples of how negative affect, i.e. HIV-related distress impacts PLWH’s well-being and physical health (e.g. Chida & Vedhara, 2009; Ickovics et al., 2006; Leserman, 2008), very little is known about the role of positive emotions in various aspects of functioning among this patient group, making this topic a candidate for further investigation (Ironson & Hayward, 2008; Moskowitz et al., 2017).

## 1. Current study

The aim of this one-year longitudinal study was to investigate the level of PTG and its relationship with social support (perceived support, need for support, actually received support) and positive and negative affect among PLWH, while also controlling for selected socio-medical covariates (see Method). In particular, a mediating role of positive and negative affect in the link between social support and PTG was explored. The following hypotheses were formulated in a longitudinal study design (Fitzmaurice, Laird, & Ware, 2011):

1. A direct and indirect positive relationship was expected between the level of social support (perceived support, need for support, actually received support) in the first assessment, and the intensity of PTG in the third assessment, while also controlling for the level of PTG in the first assessment.
2. It was expected that positive and negative affect would partially mediate the relationship described by the first hypothesis.

**Figure 1.**
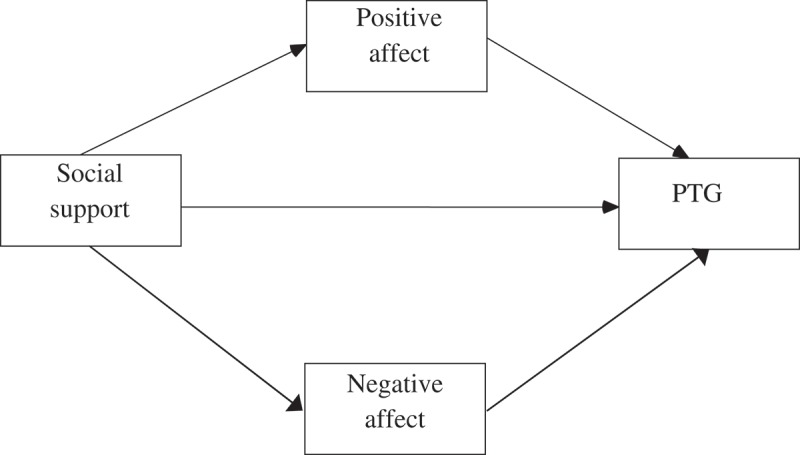
Preliminary model of the relationship between PTG, positive and negative affect and social support.

Based on the aforementioned hypotheses, a preliminary path model was constructed (see Figure 1).

## 2. Method

### 2.1. Participants

The study was conducted on patients at the Hospital of Infectious Diseases in Warsaw. The participants filled out a paper-and-pencil version of the questionnaire and participated in the study voluntarily, as there was no remuneration for participation. The eligibility criteria encompassed participants 18 years or older, a confirmed medical diagnosis of HIV infection and receiving care from the hospital where the study was conducted. The exclusion criteria included HIV-related cognitive disorders, which were screened by psychiatrists working at this hospital. The research project was accepted by the ethics committee.

The first assessment was performed between June and July 2016. A total of 129 participants were recruited to take part in the longitudinal project, i.e. those patients not only agreed to complete the questionnaires and left their contact details (i.e. telephone number and/or email address) so that the author of the study could contact them for the subsequent assessments, but they also highlighted in the Posttraumatic Growth Inventory (see, Measures) that the diagnosis of HIV infection was a traumatic event for them. The second assessment was performed between January and February 2017. Out of 129 participants from the first wave, 106 participated in the second assessment. Finally, the last assessment was conducted between May and June 2017, and 82 participants (63.6%) out of the initial sample of 129 participated in all three assessments. No missing data in the final sample of 82 participants was observed. Table 1 presents the socio-medical data in the final sample of 82 study participants and in the group of participants who did not participate in three assessments (drop-out).

**Table 1.** Socio-medical variables in the studied final sample (*N* = 82) and in the drop-out group (*N* = 47).

Variable	Drop-out (*N* = 47)	Final sample (*N* = 82)	χ^2^/T	*df*	*p*
Sex			χ^2^ = .01	1	.968
Male	40 (85.1%)	70 (85.4%)			
Female	7 (14.9%)	12 (14.6%)			
Age (in years)			T = -.62	127	.539
Range	19–58	21–76			
(*M* ± *SD*)	39.32 ± 8.42	40.50 ± 11.47			
Relationship status			χ^2^ = .24	1	.623
Stable relationship	26 (55.3%)	49 (59.8%)			
Lack of stable relationship	21 (44.7%)	33 (40.2%)			
Education			χ^2^ = 3.58	2	.167
Elementary	7 (14.9%)	5 (6%)			
Secondary	17 (36.2%)	26 (31.7%)			
University degree	23 (48.9%)	51 (62.3%)			
Employment			χ^2^ = 4.26	2	.119
Full employment	38 (80.9%)	53 (64.6%)			
Unemployment	6 (12.8%)	23 (28.1%)			
Retirement	3 (6.4%)	6 (7.3%)			
HIV/AIDS status			χ^2^ = 1.73	1	.189
HIV+ only	42 (89.4%)	66 (80.5%)			
HIV/AIDS	5 (10.6%)	16 (19.5%)			
HIV infection duration in years			T = -.68	127	.498
Range	1–27	1–30			
(*M* ± *SD*)	6.64 ± 6.58	7.39 ± 5.72			
Antiretroviral Treatment (ART) duration in years			T = -.90	127	.369
Range	1–22	1–21			
(*M* ± *SD*)	4.96 ± 4.77	5.76 ± 4.88			
CD4 Count			T = −1.93	127	.056
Range	200–1200	200–2000			
(*M* ± *SD*)	559.04 ± 225.24	645.73 ±256.23			

*M* = Mean; *SD *= Standard Deviation; T = value of Independent samples *t*-test; df = degrees of freedom; *p* = Statistical Significance for Independent samples *t*-test for Interval Scales or Pearson’s χ^2^ Test For Categorical Variables.

There were no statistically significant differences between the two samples in terms of socio-medical variables. There were also no statistical differences between the two samples in terms of psychological variables (Table 2).

### 2.2. Measures

To measure the intensity of PTG, a Polish adaptation of the Posttraumatic Growth Inventory (PTGI; Tedeschi & Calhoun, 1996) was used. It is important to underline that although the original PTGI comprises five specific domains of PTG (‘relating to others’, ‘new possibilities’, ‘personal strength’, ‘spiritual change’ and ‘appreciation of life’), the Polish adaptation of the PTGI assesses only four domains of PTG. Exploratory and confirmatory factor analysis revealed a four-factor structure for the PTG, including changes in self-perception (‘perceiving new possibilities, feeling of personal strength’), changes in relationships with others (‘feelings of greater connection with other people, increase in empathy, altruism’), greater appreciation for life (‘changes in life philosophy and current life goals, greater appreciation for every day’) and spiritual changes (‘better understanding of spiritual issues, increase in religiousness’). In the PTGI, participants rate 21 positive statements that describe various changes resulting from traumatic or highly stressful events, which are mentioned at the beginning of the inventory. The participants were instructed to focus on their HIV infection as the example of a traumatic event. The statistical analysis is usually performed only for the global PTG score (sum of all items), as particular subscales in the Polish version of PTGI are highly intercorrelated (Ogińska-Bulik & Juczyński, 2010). In addition, according to Park and Helgeson (2006), the aforementioned unifactorial assessment of PTG represents a more valid way to measure PTG compared to analysing various dimensions of growth, which may vary from study to study, even when researchers use the same PTG questionnaire. Cronbach’s α in the studied final sample at the third assessment for the whole scale was α = .86 and, for the four subscales, it varied between .81 and 85.

Social support was assessed with Schulz and Schwarzer’s (2003) Berlin Social Support Scales (BSSS), adapted in Polish by Łuszczyńska, Kowalska, Mazurkiewicz, and Schwarzer (2006). It evaluates a broad range of support dimensions and, in this study, three social support scales were used: perceived support, need for support and actually received support. The psychometric properties of the Polish version of the BSSS have been assessed on various groups of patients, including those having undergone bypass operations or following heart attacks, and patients with chronic, degenerative spinal diseases (Łuszczyńska et al., 2006). These studies have thus resulted in the confirmation of satisfactory reliability and validity. Cronbach’s α reliability coefficients for all scales in the final sample at the third assessment were satisfactory, ranging between .83 and .87.

In order to assess positive and negative affect, a Polish adaptation (Brzozowski, 2010) of the PANAS-X was used (Watson, Clark, & Tellegen, 1988). The PANAS-X comprises 10 adjectives for positive affect (e.g. *proud, excited*, etc.) and 10 for negative affect (e.g. *frightened, hostile*, etc.). The participants were asked to rate their general affective states on a five-point response scale from 1 (*not at all*) to 5 (*extremely*). The Cronbach’s alpha coefficients in the studied final sample at the third assessment were .81 for the positive affect subscale and .83 for the negative affect subscale.

Socio-medical covariates were assessed via separate survey. More specifically, participants’ age, CD4 count and antiretroviral treatment duration were continuous variables. Participants’ sex, relationship status and HIV/AIDS status were dichotomous variables. Finally, employment and education were categorical variables. All the categories of these covariates are described in Table 1.

## 3. Data analysis

The data analysis consisted of three stages and was conducted on the final sample of 82 participants. Each variable was measured three times. First, possible differences between the three assessments were examined. With the use of repeated measures ANOVA followed by multiple comparisons performed with Bonferroni correction, changes in the analysed variables in time were shown. Possible changes in three time points were analysed in all examined variables. Second, control variables were selected from the socio-medical data. Multiple regression analysis via the stepwise method was used to select statistically significant covariates for the analysed variables. There were six regression models, a separate regression model for each analysed variable. Finally, path analysis was performed to verify the preliminary model depicted in Figure 1, with consideration of selected socio-medical data. Each type of social support (perceived support, need for support, actually received support) was analysed in a separate model. In the statistical analysis, the IBM SPSS 24 and IBM AMOS 24 statistical packages were used (IBM Corp. Released, 2016).

## 4. Results

The results of changes in the three assessments with respect to the variables in the PTGI, PANAS and BSSS are presented in Table 3. There were no statistically significant changes across the three assessments with respect to all analysed variables. Table 3 presents values of Kolmogorov-Smirnov normality test. None of the analysed variables significantly differed from the normal distribution (Table 4).

**Table 2.** Mean values of PTGI, PANAS and BSSS in the studied final sample (*N* = 82) and in the drop-out group (*N* = 47).

	Mean (*SD*)		Independent samples *t*-test	
	Drop-out (*N* = 47)	Final sample (*N* = 82)	*t*^a^	*p*
PTG	58.91 (24.56)	61.24 (22.19)	−.55	.582
Positive Affect	3.47 (0.61)	3.41 (.69)	.51	.613
Negative Affect	2.49 (0.98)	2.25 (.92)	1.35	.179
Perceived Support	19.30 (4.77)	18.65 (5.33)	.69	.489
Need for Support	7.17 (2.97)	7.36 (2.38)	−.38	.702
Actually Received Support	30.94 (9.13)	29.48 (10.31)	.81	.421

*SD* = Standard Deviation; *t* = Value of independent samples *t*-test;

*p* = Statistical Significance; a = *df* (Degrees of Freedom) = 127.

**Table 3.** Changes in three assessments with respect to variables in the PTGI, PANAS and BSSS with results of repeated measures ANOVA.

	Mean (*SD*)			p*			
	T1	T2	T3	Overall	T1 vs. T2	T2 vs. T3	T1 vs. T3
PTG	61.24 (22.19)	65.39 (24.91)	63.51 (22.25)	*F*(2,162) = 1.53	.280	.999	.999
	*Z* = .52	*Z* = .95	*Z* = 1.00	*p* = .220			
Positive Affect	3.41 (.69)	3.39 (0.80)	3.33 (.69)	*F*(2,162) = .51	.999	.932	.999
	*Z* = .72	*Z* = .52	*Z* = .91	*p* = .603			
Negative Affect	2.25 (.92)	2.17 (0.84)	2.23 (.93)	*F*(1.87,151.03) = .26	.999	.999	.999
	*Z* = 1.30	*Z* = 1.34	*Z* = 1.02	*p* = .755			
Perceived Support	18.65 (5.33)	19.17 (5.04)	18.68 (5.49)	*F*(2,162) = .48	.999	.999	.999
	*Z* = 1.03	*Z* = 1.06	*Z* = 1.05	*p* = .621			
Need for Support	7.36 (2.38)	7.24 (2.92)	7.15 (2.77)	*F*(2,162) = .22	.999	.999	.999
	*Z* = 1.21	*Z* = 1.11	*Z* = 1.08	*p* = .804			
Actually Received Support	29.48 (10.31)	31.57 (10.94)	31.85 (10.29)	*F*(2,162) = 2.11	.331	.237	.999
	*Z* = 1.08	*Z* = 1.25	*Z* = .91	*p* = .124			

*SD* = Standard Deviation; T1 = First Assessment; T2 = Second Assessment; T3 = Third Assessment; *Z* = value of Kolmogorow-Smirnow normality test; * = *p* Values Of Repeated Measures Analyses Of Variance and Bonferroni’s Multiple Comparisons.

Second, the control variables were selected from the socio-medical data. It is important to note that there were no changes over the three time points for relationship status, education, employment and HIV/AIDS status. In addition, frequency distribution of education showed that the three education categories (elementary, secondary, university degree) were very unequal in terms of number of participants. Very few participants had only elementary education. Thus, it was decided to merge the two groups of participants with elementary and secondary level of education to achieve better statistical power for detecting any relationships between education and explanatory variable. The same procedure was applied to three categories of employment variable. Unemployed and retired participants were merged into one group due to the small number of participants in both categories and in order to achieve better statistical power.

There were positive relationships between higher education and perceived support and between higher education and received support. The participants who were not in stable relationships reported lower received support. Women had a significantly higher need for support than men. There were also statistically significant negative relationships between HIV/AIDS status and positive affect in the second assessment and PTG in the third assessment, and between negative affect and being employed in the second assessment.

Finally, AMOS graphics was used to create path models to verify the relationships depicted in the preliminary model in Figure 1 (Hooper, Coughlan, & Mullen, 2008; Kline, 2015). Correlations between analysed variables are depicted in Table 5.

**Table 4.** Results of multiple regression analysis. Selection of control variables out of socio-medical data.

	Assessment	Predictor	*B*	*Beta*	*t*	*p*	*ΔF*	*df*	*p*	*ΔR^2^*
Perceived Support	First	Higher Education	4.03	.37	3.56	.001	12.70	1,80	.001	.14
Need For Support	First	Sex	1.74	.24	2.23	.028	4.98	1,80	.028	.06
Actually Received Support	First	Lack Of Stable Relationship	−5.53	−.27	−2.57	.012	8.74	1,80	.004	.10
		Higher Education	4.87	.24	2.24	.028	5.03	1.79	.028	.05
Positive Affect	Second	HIV/AIDS Status	−.55	−.29	−2.73	.008	7.45	1.80	.008	.09
Negative Affect	Second	Employment	−.45	−.25	−2.31	.024	5.33	1.80	.024	.06
PTG	Third	HIV/AIDS Status	−18.17	−.35	−3.37	.001	11.37	1.80	.001	.12

**Table 5.** Correlations between analysed variables included in path analysis.

Variables	Assessment	1.	2.	3.	4.	5.	6.
(1) Perceived Support	First	–	.484**	.672**	.221*	−.276*	.140
(2) Need For Support	First		–	.478**	.360**	−.088	.302**
(3) Actually Received Support	First			–	.254*	−.155	.116
(4) Positive Affect	Second				–	.012	.292**
(5) Negative Affect	Second					–	−.019
(6) PTG	Third						–

*p < .05; **p < .01.

**Figure 2.**
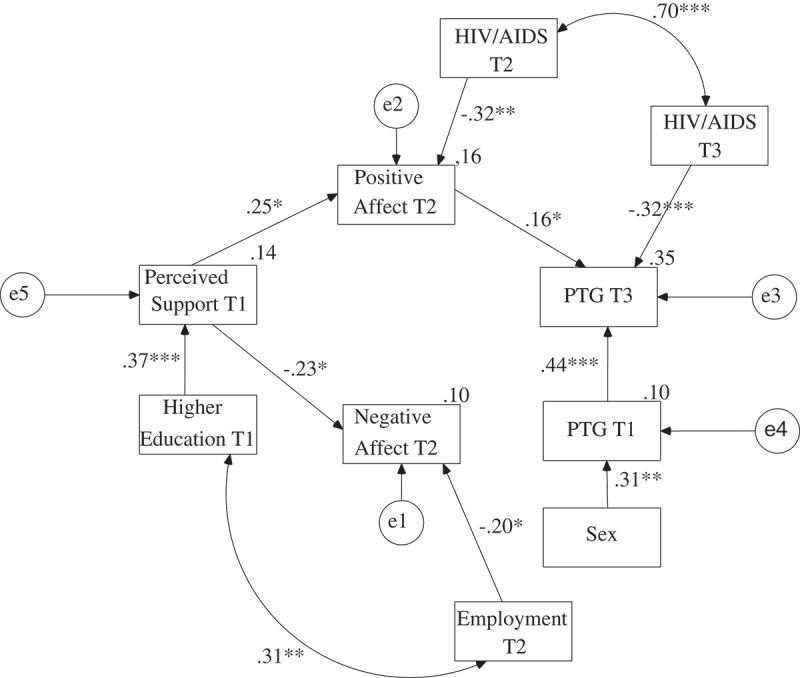
The final model of the relationship between PTG and perceived support, positive and negative affect with regression coefficients. *Note*. T1 = First Assessment; T2 = Second Assessment; T3 = Third Assessment.*p < .05; **p < .01; ***p < .001.

**Figure 3.**
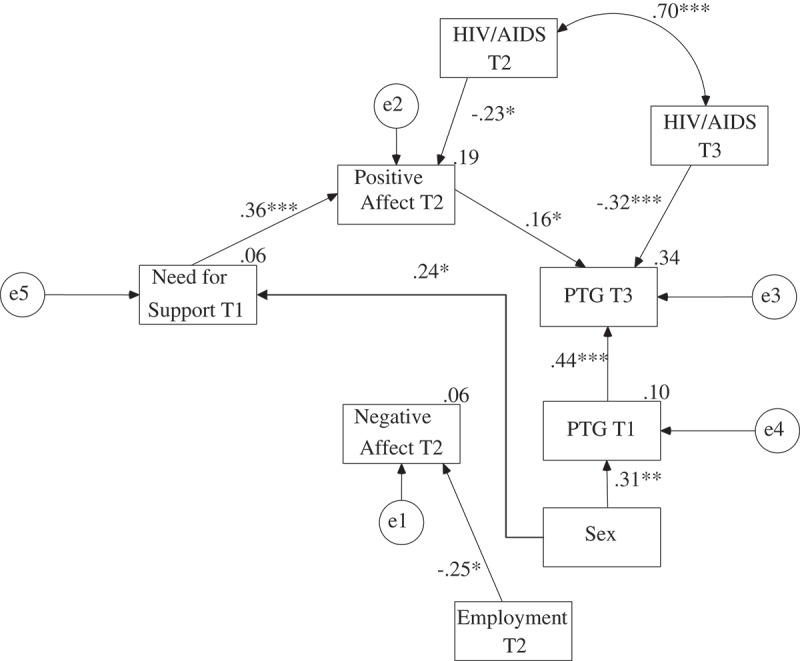
The final model of the relationship between PTG and need for support, positive and negative affect with regression coefficients. *Note*. T1 = First Assessment; T2 = Second Assessment; T3 = Third Assessment.*p < .05; **p < .01; ***p < .001.

**Figure 4.**
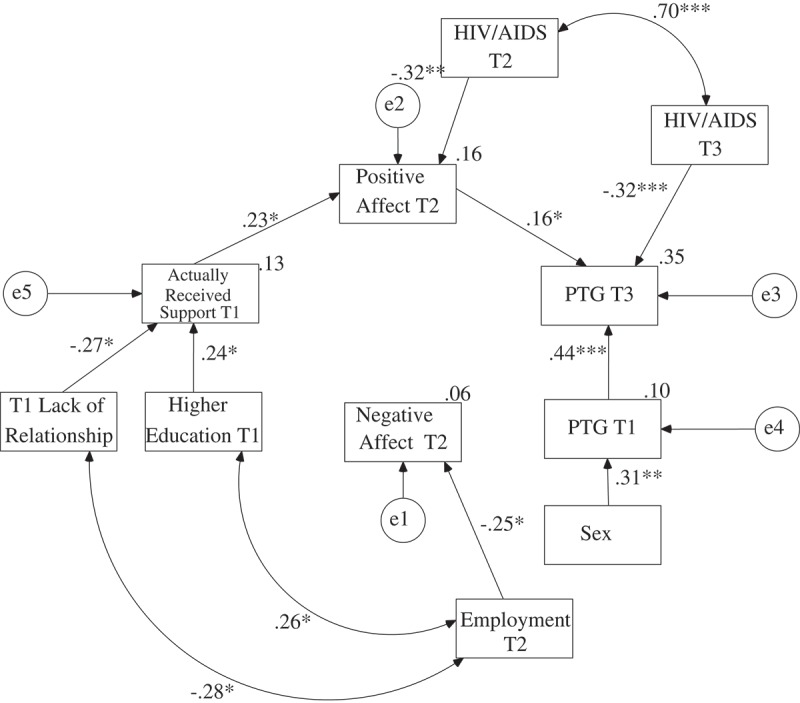
The final model of the relationship between PTG and actually received support, positive and negative affect with regression coefficients. *Note*. T1 = First Assessment; T2 = Second Assessment; T3 = Third Assessment.*p < .05; **p < .01; ***p < .001.

The maximum likelihood method was used to create the path model. Perceived support, need for support and received support were analysed in separate models as exogenous variables. The global PTG level in the third assessment was analysed as the explained variable. The global PTG in the first assessment was controlled in all models. Contrary to the preliminary hypotheses, the paths between negative affect and PTG were statistically insignificant in all three models. Moreover, there were no direct relationships between perceived support, need for support, received support and PTG. There were also no relationships between need for support, received support and negative affect. Therefore, these paths were eliminated from the models. The final models are presented in Figure 2, 3 and 4.

Figure 2 presents the final model for perceived support. The values of the goodness of fit indices suggest that the model was very satisfactory, χ^2^(34) = 38.43, *p *> .05; CFI = .97; RMSEA = .04; AGFI = .87. The final model explained 34.7% of the variance of the global PTG level in the third assessment. Perceived support in the first assessment had a positive impact on positive affect in the second assessment, and positive affect in the second assessment had a positive impact on PTG in the third assessment when controlling for PTG in the first measurement. Thus, there was full mediation between perceived support in the first assessment and PTG in the third assessment via positive affect in the second assessment. The value of one-tailed Sobel test calculated on regression coefficients acquired in the model in which negative affect was also controlled for was statistically significant, *Z* = 1.81, *p* < .05. Perceived support in the first assessment was also negatively related to negative affect in the second assessment. Higher education was positively related to perceived support in the first assessment. Employment was negatively related to negative affect in the second assessment. HIV/AIDS status was negatively related to positive affect in the second assessment and to PTG in the third assessment. There was a positive relationship between higher education and employment. Women had higher PTG in the first assessment than men.

Figure 3 presents the final model for need for support. The values of the goodness of fit indices suggest that this model was also very satisfactory, χ^2^(27) = 20.65, *p* > .05; CFI = .99; RMSEA = .01; AGFI = .91. The final model explained 34.5% of the variance of the global PTG level in the third assessment. Need for support in the first assessment also had a positive impact on positive affect in the second assessment, and positive affect in the second assessment had a positive impact on PTG in the third assessment when controlling for PTG in the first assessment. Thus, there was full mediation between need for support in the first assessment and PTG in the third assessment via positive affect in the second assessment. The value of one-tailed Sobel test calculated on regression coefficients acquired in the model in which negative affect was also controlled for was statistically significant, *Z* = 1.83, *p* < .05. Need for support in the first assessment was not related to negative affect in the second assessment. Employment was negatively related to negative affect in the second assessment. HIV/AIDS status was negatively related to positive affect in the second measurement and to PTG in the third measurement. Women had a significantly higher need for support than men and higher levels of PTG in the first assessment than men.

Figure 4 presents the final model for received support. The values of the goodness of fit indices suggest that this model was also very satisfactory, χ^2^(43) = 46.29, *p* > .05; CFI = .98; RMSEA = .03; AGFI = .86. The final model explained 34.6% of the variance of the global PTG level in the third assessment. Received support in the first assessment also had a positive impact on positive affect in the second assessment, and positive affect in the second assessment had a positive impact on PTG in the third assessment when controlling for PTG in the first assessment. Thus, there was full mediation between received support in the first assessment and PTG in the third measurement via positive affect in the second assessment. The value of one-tailed Sobel test calculated on regression coefficients acquired in the model in which negative affect was also controlled for was statistically significant, *Z* = 1.85, *p* < .05. Received support in the first assessment was not related to negative affect in the second assessment. Higher education was positively related to received support in the first assessment, while lack of a stable relationship was negatively related to received support in the first measurement. Employment was negatively related to negative affect in the second assessment. HIV/AIDS status was negatively related to positive affect in the second assessment and to PTG in the third assessment. Lack of a stable relationship in the first assessment was negatively related to employment in the second assessment. There was also a positive relationship between higher education and employment. Women had higher levels of PTG in the first assessment than men.

## 5. Discussion

The aim of this one-year longitudinal study was to investigate the level of PTG and its relationship with social support dimensions and positive and negative affect among PLWH. In particular, this study explored the mediating role of positive and negative affect in the link between social support and PTG. The first hypothesis was partially confirmed, as only an indirect link was observed between all examined social support dimensions and the level of PTG among the participants. Similarly, the second hypothesis was also somewhat confirmed, as positive affect, but not negative affect, completely mediated the aforementioned relationship. It seems that the study participants who perceived, received or had higher needs of social support at the baseline experienced more positive emotions over time, which in turn was positively related to the level of PTG in the third assessment. This result may indicate an important mechanism linking social support to PTG in the aftermath of life threatening illness, especially that the nature and direction of this relationship is ambiguous (e.g. Cieslak et al., 2009; Rzeszutek et al., 2017; Sheikh, 2004). As noted earlier in Tedeschi and Callhoun’s (2004) model, social support facilitates PTG by mobilizing cognitive processing after trauma; it also stimulates meaning searching after trauma. At the same time, in the aforementioned theoretical model, adequate emotion regulation, especially maintaining a high level of positive affect, is crucial for meaning searching after trauma and in facilitating growth. More specifically, Boyraz and Efstathiou (2011) observed that positive affect mediated the link between self-focusing tendencies (ruminations), meaning in life and PTG among bereaved women. Some researchers even highlighted common neural mechanisms of PTG and positive affect, i.e. increased left frontal brain activity (Rabe, Zöllner, Maercker, & Karl, 2006). Conversely, the lack of a relationship between social support, PTG and negative affect was intriguing, as high levels of negative affect occurred to hinder PTG in some studies (Boyraz et al., 2010). Nevertheless, some researchers observed that PTG is associated only with a greater level of positive affect, but not with negative affect (Schroevers et al., 2010; Yu et al., 2014).

In discussing the aforementioned results in the context of PLWH, it should be noted that numerous researchers observed that social support not only enhances well-being, i.e. protects from depression and an improves social status (Lee, Detels, Rotheram-Borus, Duan, & Lord, 2007), but also impacts health outcomes, i.e. reduces HIV-related physical symptoms (Ashton, Vosvick, & Chesney, 2005). In addition, as noted earlier, the literature on PLWH has focused predominantly on the negative aspects of HIV/AIDS, so the knowledge about positive factors, including positive emotions among this patient group, is very scarce. In one of the few studies on this topic, Moskowitz’s (2003) seven-year longitudinal study found lower mortality among HIV-infected men with higher levels of positive affect. Similarly, Wilson et al. (2016), in a national study of HIV-infected women, observed that positive affect predicted viral suppression, which may also be understood that viral suppression can affect positive affect and generate a cycle of improvement for PLWH. Moreover, positive affect among PLWH was negatively related to the level of depression (Li, Mo, Wu, & Lau, 2016) and slower HIV progression (Ironson & Hayward, 2008), and this effect was independent of negative affect. Employing Fredrickson’s (2001) broaden-and-build theory – in which positive emotions my enhance personal resources, well-being and psychological growth over time – Yu et al. (2014) claimed that positive affect should be treated as one of the most important predictors of PTG, especially in cases of life threatening diseases.

Finally, the results of this study demonstrate that, from the medical data, only HIV/AIDS status was negatively related to the level of PTG and the intensity of positive affect. Social support was related only to sociodemographic data, such as employment, higher education and a stable relationship, and not with medical data. This corresponds to other studies, which proved that an especially high threat of social rejection exists among PLWH, including deteriorating social status (Samson, Lavigne, & MacPherson, 2009). Notwithstanding, this finding may be interpreted in light of the great progress in HIV treatment, which resulted in HIV/AIDS being perceived as more of a chronic than a terminal condition; thus, psychosocial factors may be more important for well-being among PLWH compared to clinical variables (Deeks, Lewin, & Havlir, 2013). However, it should be noticed also that the study sample consists of highly functional PLWH with a CD4 level comparable to that of the general population (EACS, 2017), which may be the reason why almost all medical variables were not related to PTG among participants. Finally, HIV-infected women revealed higher levels of PTG, which correspond to studies conducted on the general population (Vishnevsky, Cann, Calhoun, Tedeschi, & Demakis, 2010) as well as among PLWH (Rzeszutek, Oniszczenko, & Firląg-Burkacka, 2016).

## 6. Strengths and limitations

This study has a few strengths, including that it is a longitudinal and theory-based study design with three assessments and that it investigates various dimensions of social support in relation to PTG. However, several limitations should also be noted. First, the final sample of the three assessments was relatively low due to high dropout. Second, for organizational reasons, the sample was heterogeneous in respect to the duration of HIV infection and, although this clinical variable was not significant in the path models, this fact ought to be reconsidered in discussing the findings of the study. Third, the PTGI questionnaire was used in this study, which measures retrospective reports of PTG. Some researchers have indicated that this is a method of assessment of growth rather than a reflect of real positive change, which may indicate some positive illusions (Frazier et al., 2009). In addition, HIV-related trauma is not a discrete traumatic event, i.e. PLWH may continue to feel traumatized by the diagnosis and healthcare regimens they are required to follow. Thus, there are authors who underlined that PTGI may not fully capture illness-related aspects of trauma and associated growth (Casellas-Grau, Ochoa, & Ruini, 2017). Finally, the PANAS-X questionnaire is not an appropriate tool for measurement emotion regulation strategies, and future research should focus on testing a similar model presented in this study while measuring emotion regulation strategies directly.

## 7. Conclusions

This study suggests the need for more research on positive aspects of HIV/AIDS, particularly PTG. Clinicians and researchers should further investigate the combined role of social support and positive affect in promoting positive change among PLWH. More specifically, complementary and integrative interventions, such as mindfulness, focused on enhancement and sustainment of positive affect among PLWH, should be an adjunct to traditional mental health screening among this patient group, especially that the effectiveness of such interventions for psychological health has been empirically proven (Moskowitz et al., 2017; Riley & Kalichman, 2015; Wilson et al., 2016).
